# Clinical and functional outcomes of vascularized bone graft in the treatment of scaphoid non-union

**DOI:** 10.1371/journal.pone.0197768

**Published:** 2018-05-22

**Authors:** Alireza Rahimnia, Amir-Hossein Rahimnia, Abdolkarim Mobasher-Jannat

**Affiliations:** 1 Trauma Research center, Department of Medicine, Baqiyatallah University of Medical Sciences, Tehran, Iran; 2 Department of Medicine, Tehran University of Medical Sciences, Tehran, Iran; 3 Chemical Injuries Research Center, Systems Biology and Poisonings Institute, Baqiyatallah University of Medical Sciences, Tehran, Iran; University of Umeå, SWEDEN

## Abstract

**Introduction:**

Scaphoid non-union is a challenging and complex problem. Various methods have been proposed for the management of patients with scaphoid non-union and to reduce the risk of complications. In this study, our aim was to evaluate the clinical and functional outcomes of using a vascularized bone graft in the treatment of scaphoid non-union.

**Methods:**

Patients with scaphoid non-union who underwent 1,2 intercompartmental supraretinacular artery pedicled vascularized bone graft between January 2005 and January 2011 were enrolled. The parameters assessed included clinical and functional outcomes, radiological measures, and potential risk factors.

**Results:**

Forty-one patients were finally included. Thirty patients achieved union (73%) and 11 did not. Smoking was a significant risk factor for non-union after the surgery. In patients who achieved union, grip strength and radioulnar abduction were greater in comparison to that in patients who did not achieve union. Functional measures, including the Disabilities of Arm and Shoulder score and the Modified Mayo Wrist Score, improved in patients with scaphoid union. The scaphoid length also improved significantly postoperatively in these patients.

**Conclusion:**

Surgical treatment of scaphoid non-union using vascularized bone graft led to a high union rate with good clinical and functional outcomes. Smoking is a risk factor for non-union, even with the use of a vascularized bone graft. Avascular necrosis was not associated with an increased risk for non-union.

## Introduction

Scaphoid fractures account for 50–80% of all carpal fractures [1, 2], with the highest incidence in young and active men [3]. According to epidemiological studies, the incidence of scaphoid fractures varies between 0.08–1.21 per 1000 person-years in different populations. These fractures usually are treated with conservative measures, resulting in union rates between 55–100% [4, 5]. Approximately 10% of scaphoid fractures progress to non-union [6], which often is the consequence of different risk factors (Table 1). Complications of neglected scaphoid non-union (SN) are summarized in Table 1 [7–13].

**Table 1:** Risk factors leading to nonunion scaphoid fractures and nonunion complications.

Risk factors for nonunion	Results of scaphoid malunion or non-union
Displacement of more than 1 mm	Pain
Fracture of the proximal pole	Altered carpal kinematics
History of osteonecrosis	Diminished range of motion
Vertical oblique fracture pattern	Disuse osteopenia
Nicotine use	Decreased grip strength
Delay in diagnosis	Dorsal intercalary segmental instability
Inadequate immobilization	Degenerative changes (scaphoid nonunion advanced collapse)

Numerous bone grafting techniques have been proposed for the treatment of SN. The differences between these techniques include the bone graft harvest site, fixation method and using vascularized bone graft (VBG) or non-vascularized bone graft (NVBG) [14–18].

VBG is a reasonable treatment option because it maintains cell viability and drives a bone healing process similar to primary fracture healing [19]. Quicker stabilization and lesser immobilization period are other advantages of VBGs [20].

Various vascularized grafts have been used previously [21–26] and the main donor sites were the palmar and dorsal parts of the radius. The method of 1,2 intercompartmental supraretinacular artery pedicled vascularized bone graft (1,2-ICSRA-VBG) was used by Zaidemberg [26] for the first time in 1991 and is the most commonly used method because of the proximity to scaphoid bone [19]. However, its outcomes were controversial because various studies demonstrated union rates between 27–100% [24, 26–30]. Therefore, it is difficult to consider a specific surgery technique as superior to others [31, 32].

The main purpose of this study was to present the clinical outcomes of scaphoid non-union (SN) treated using 1,2-ICSRA-VBG.

## Patients and methods

### Patient data

A retrospective study was designed to evaluate the records of patients with SN between January 2005 and January 2011. Patients with SN diagnosed by X-ray or computed tomography (CT) who were treated with 1,2-ICSRA-VBG and had postoperative follow-up with X-ray or CT were included. Ethics approval was obtained from the Baqiyatallah hospital ethics committee. All patients signed an informed consent allowing us to use their medical records in the study. The surgery technique used has been previously reported [24, 26]. Avascular necrosis (AVN) was diagnosed intraoperatively by the absence of punctate bleeding at the non-union site.

Radiographic examinations were performed at 6, 12, and 16 weeks after the surgery and during the latest visit. Different X-ray views (posterior-anterior, lateral, and scaphoid) were obtained and CT was used if X-ray was unclear for diagnosing union.

Immobilization using a cast was performed for all patients at least for 6 weeks. Subsequently, the cast was removed if union was achieved. After 16 weeks, the cast was discontinued regardless of achievement of union.

Demographic characteristics and baseline data of patients were obtained from their medical records. Postoperative data were collected at the end of the follow-up period.

We assessed the scaphoid length (mm), scapho-lunate angle (degree), Nattrass Carpal Height Index, active range of motion (degree), Jamar grip strength (kg), key pinch strength (kg), and tripod pinch strength (kg) for clinical assessment. For evaluating clinical outcomes, we used the Disabilities of Arm and Shoulder (DASH) questionnaire (0: no limitation, 100: maximum limitation) and the Modified Mayo Wrist Score (MMWS; 91–100: excellent; 80–90: good; 65–79: fair; and < 65: poor).

### Statistical analysis

Student’s t test or Wilcoxon rank sum test was used to analyze normally distributed continuous variables. We compared categorical variables using chi-square or Fisher’s exact test. To identify the risk factors affecting DASH score more than 50 and MMWS less than 65, binary logistic regression analysis was performed. P<0.05 was considered significant and all calculations were performed using SPSS software, version 22 (IBM Corp., Armonk, NY, USA).

## Results

Fifty-four patients underwent 1,2-ICSRA-VBG. Thirteen patients were lost to follow-up, and forty-one patients satisfied the inclusion criteria. The demographic data and characteristics are summarized in Table 2. The total number of subjects included 35 males and 6 females with a mean age of 26.7±7 years (mean ± standard deviation, SD). The mean body mass index (BMI) was 22 kg/m^2^ and 19 patients were current smokers. Thirty-three patients had proximal pole fractures and none of the patients underwent bone grafting before vascular bone grafting. The mean delay between scaphoid fracture and vascular surgery for the treatment of non-union was 47 (±11) months and the mean duration of the surgery was 100±22 minutes. Twenty-six patients were diagnosed with AVN intra-operatively.

**Table 2:** Clinical characteristics of patients and their association with postoperative outcomes.

Measure, n (%)		Total (n = 41)	Non-union (n = 11)	Union (n = 30)	*P* value
**Age, years**	>25 y	19 (46%)	7 (64%)	12 (40%)	0.30
	≤25 y	22 (54%)	4 (36%)	18 (60%)	
**Sex**	Female	4 (10%)	3 (27%)	1 (3%)	0.25
	Male	37 (90%)	8 (73%)	29 (97%)	
**Smoking**	Yes	19 (46%)	9 (82%)	10 (33%)	0.02
	No	22 (54%)	2 (18%)	20 (67%)	
**BMI, kg/m**^**2**^	>22	18 (44%)	6 (55%)	12 (40%)	0.45
	≤22	23 (56%)	5 (45%)	18 (60%)	
**Time to surgery, months**	>47	16 (39%)	6 (55%)	10 (33%)	0.33
	≤47	25 (61%)	5 (45%)	20 (67%)	
**PIN neurectomy**	Yes	15 (37%)	3 (27%)	12 (40%)	0.80
	No	26 (63%)	8 (73%)	18 (60%)	
**K-wire fixation**	Yes	10 (24%)	2 (18%)	8 (27%)	0.59
	No	31 (76%)	9 (82%)	22 (73%)	
**Avascular necrosis**	Yes	26 (63%)	6 (55%)	20 (67%)	0.73
	No	15 (37%)	5 (45%)	10 (33%)	
**Follow-up**	>49	23 (56%)	8 (73%)	15 (50%)	0.66
	≤49	18 (44%)	3 (27%)	15 (50%)	

The follow-up period was 49±21 months. Thirty patients achieved union and eleven did not. The diagnosis of non-union was based on X-ray taken at 4 and 6 weeks intervals; therefore, we were unable to recognize the exact time of union.

The overall mean DASH score and mean MMWS score were 26 and 78, respectively, at mean follow-up of 49 months postoperatively. All patients with successful union returned to their previous jobs without any limitation. Of the patients who did not achieve union after the surgery, four kept their previous jobs, four lost their jobs, and three changed their occupation.

Outcomes of patients who achieved union at the end of follow-up are summarized in Table 3. The MMWS score improved from 60 preoperatively to 83 at the last follow-up. Excellent results were achieved in 14 patients (46.6%), good results in 10 (33.3%), fair results in 5 (16.6%), and poor results in only 1 (3.5%). The DASH score decreased from 54 to 21, and grip strength decreased to 73% of the contralateral hand strength.

**Table 3:** Analysis of preoperative and postoperative outcomes of patients who achieved scaphoid union.

	Pre-operation	Post-operation	Contralateral	% contralateral
***AROM (***^***◦***^ ***)***				
Extension-flexion	103±11	101±16	125±20 *	81%
Radio-ulnar abduction	43±17	53±19 †	77±12 *	69%
***Clinical and Functional Evaluation***				
DASH Score	54±22	21±17 †	------------	------------
MMWS Score	60±16	83±9 †	------------	------------
Jamar Grip Strength (kg)	------------	30±10	41±12 *	73%
Key Pinch Strength (kg)	------------	10±2	11±2	91%
Tripod Pinch Strength (kg)	------------	9±4	9±4	100%
***Radiologic Measurements***				
Scaphoid Length	20±2	23±3 †	------------	------------
Scapho-lunate angle (^◦^)	56±7	57±5	------------	------------
Nattrass Carpal Height Index	1.55±0.12	1.56±0.02	------------	------------

*P < .05 when comparing with postoperative values.

^†^P < .05 when comparing with preoperative values.

Radio-ulnar deviation pre- and post-operatively improved significantly but extension-flexion did not show significant differences. With comparison to the contralateral wrist, there was significant limitation of both radio-ulnar deviation and extension-flexion movements. During radiological evaluation, scapho-lunate angle and Nattrass carpal height index did not present any significant difference. Scaphoid length increased significantly after the operation.

Differences of outcomes between patients who achieved union and who did not are presented in Table 4. Of the reported outcomes, radio-ulnar deviation and grip strengths had significantly improved in patients with scaphoid union.

**Table 4:** Comparison of outcomes between patients who achieved union and those who did not.

	Union (n = 30)	Non-union (n = 11)	*P* Value
***AROM (***^***◦***^ ***)***			
Extension-flexion	101±16	95±10	0.2
Radio-ulnar abduction	53±19	33±13	0.03
***Clinical and Functional Evaluation***			
Jamar Grip Strength (kg)	30±10	19±8	0.04
Key Pinch Strength (kg)	10±2	9±3	0.4
Tripod Pinch Strength (kg)	9±4	9±1	0.6

After exploration for risk factors affecting postoperative non-union, only smoking was identified as a risk factor (Table 2). After performing logistic regression analysis, we did not find any risk factors for MMWS score less than 65 and DASH score more than 50.

## Discussion

Scaphoid nonunion is a challenging and complex problem which results owing to multiple factors. Review of literature demonstrates union rates of 74–84% using non-vascularized bone grafts [32, 33] and there is no superiority of bone autografts harvested from the iliac crest or of those from distal radius according to the morbidity [34].

Fracture healing is a multi-step process that is facilitated by the interaction of different cellular elements to achieve union. Theoretically, the use of VBGs in non-union fractures provides essential components such as cytokines and cellular mediators by maintaining cell viability, which drives the healing process similar to the primary process of fracture healing [19].

Vascularized bone grafts were introduced more than two decades ago and became popular in the treatment of SN [24, 26, 29, 30, 32, 33, 35–40]. Different studies have reported different union rates from 27–100% [29, 30].

Reviewing previous studies that have compared VBG and NVBG in the treatment of SN has revealed heterogeneous outcomes. Two prospective studies by Ribak et al. [41] and Caporrino et al. [42] demonstrated better healing rates and quicker bone healing, respectively, with VBG in comparison with NVBG. A recent systematic review and meta-analysis of 1602 patients reported 92% union rate with the use of VBG compared with 88% union rate with NVBG [32]. Patient selection appears to be an important factor influencing the union rates [43].

In our study, 41 patients treated with 1,2-ICSRA-VBG were included. Similar to most studies in this regard, none of the patients had undergone previous NVBG transplantation. However, Hirch et al. [43] and Werdin [44] reported that previous iliac crest bone graft was not a risk factor for non-union after VBG transplantation surgery.

Previous studies reported AVN as a risk factor for non-union [33, 45] and Chang et el. [46] reported better union rates without AVN. Our study included 26 patients with AVN; 20 went on to achieve scaphoid union and 6 did not. However, AVN was not found to have a significant impact on union rates, in concordance with the findings of Malizos et al. [29] and Tsai et al. [24]. A meta-analysis of 1827 scaphoid non-union repairs also reported superiority of VBG in patients with AVN [33].

We found smoking to be a significant risk factor for non-union in agreement with the results of previous studies [46, 47]. Smoking appears to be the main negative prognostic factor affecting the achievement of union in patients. Smoking impairs angiogenesis [48] and has a negative effect on fracture union [49]. Al-Hadithy et al. [49] also concluded that smoking has more negative effect on the union of fractures that require bone grafts and increases the chance of devascularization after the grafting is performed.

Previous studies have reported varying results regarding grip strength after the achievement of union [26, 29, 35, 36, 47, 50]. In our study, the grip strength of the injured hand was 73% that of the contralateral hand, which was significantly lower. Therefore, our results are in agreement with those of Malizos et al [29] and Hirche et al [43]. We observed significant improvements in grip strength of patients who achieved union than in those who did not. Therefore, we can conclude that the achievement of union restores grip strength.

Our study demonstrated that active radioulnar deviation was significantly increased in hands with scaphoid union, and compared to that of hands with non-union, it was significantly higher. Our results are contradictory to those of Malizos et al. [51].

In our study, of the patients with scaphoid union, the scapho-lunate angle and Nattrass carpal height ratio did not change significantly after the surgery, which is in accordance with the results of Malizos et al. [29], Steinmann et al. [40], and Hirche et al. [43]. However, Malizos et al. [51] reported in a long-term study of SN treated with VBG that both scapho-lunate angle and Nattrass carpal height ratio returned to normal after five years. According to our results, the length of scaphoid improved significantly after the surgery. Normalization of scaphoid length retains the tension of the tendons crossing the wrist and thus improves grip strength.

The DASH score was significantly decreased and the MMWS score was significantly increased, thus reflecting overall improvement of clinical and functional outcomes after the surgery. A long-term study of scaphoid surgeries for non-union demonstrated positive effects in terms of MMWS, DASH, and VAS with early surgery before the progression to arthritic changes [52]. Deformity correction and achievement of anatomical union are critical to achieving stable and long-lasting wrist function. However, our study was limited in determining the correlation between the achievement of anatomical union and better clinical outcomes.

The limitations of our study are as follows. First, we lost 13 patients to follow-up, and considering the relatively small sample size of our study, our results may be biased because of the missed patients and may not be representative of the entire population. Second, we were unable to exactly determine the time of union. Finally, we could not evaluate the revascularization of scaphoid bone after the surgery.

In conclusion, we found that surgical treatment of scaphoid non-union using vascularized bone graft led to a high union rate with good clinical and functional outcomes; smoking is a risk factor for non-union, even with the use of a vascularized bone graft; and that avascular necrosis was not associated with an increased risk for non-union.
